# Preparation and Evaluation of Complexed Ubiquinone (Coenzyme Q10) Antiaging Hyaluronic Acid–Vitamin C Serum for Skin Care

**DOI:** 10.1111/jocd.16706

**Published:** 2024-12-30

**Authors:** Hawazin Arkan Yousif, Israa Al‐Ani, Maha N. Abu Hajleh, Sina Matalqah, Wael Abu Dayyih, Emad A. Al‐Dujaili

**Affiliations:** ^1^ Department of Pharmaceutics and Pharmaceutical Technology, Pharmacological and Diagnostic Research Center Faculty of Pharmacy, Al‐Ahliyya Amman University Amman Jordan; ^2^ Department of Cosmetic Science, Pharmacological and Diagnostic Research Centre, Faculty of Allied Medical Sciences Al‐Ahliyya Amman University Amman Jordan; ^3^ Department of Pharmaceutical Chemistry, Faculty of Pharmacy Mutah University Al‐Karak Jordan; ^4^ Queen's Medical Research Institute, Centre for Cardiovascular Science University of Edinburgh Edinburgh UK

**Keywords:** antiaging, coenzyme Q10, hyaluronic acid, skin care, ubiquinone

## Abstract

**Background:**

Coenzyme Q10 (CoQ10) is widely recognized for its powerful antioxidant properties, sparking considerable interest in its application within skincare treatments. However, its inherently poor water solubility has posed a major challenge in formulating effective skincare products.

**Methods:**

This research aimed to develop and evaluate a water‐soluble CoQ10 serum by forming a complex with hydroxypropyl β‐cyclodextrin (HPβCD). The study focused on assessing its physicochemical properties, CoQ10 concentration, spread ability, viscosity, pH, physical stability, irritation potential, and diffusion performance. The complexation process was carried out using kneading and trituration techniques, with thorough characterization via validated analytical methods such as solubility tests, Fourier transform infrared spectroscopy (FTIR), and differential scanning calorimetry (DSC) analyses.

**Results:**

The CoQ10–HPβCD complex prepared using the trituration technique at a 2:1 ratio (CoQ10 to HPβCD) demonstrated superior water solubility, reaching 17.5 ± 1.8 mg mL^−1^, the highest among the tested formulations. Moreover, this formulation achieved the greatest encapsulation efficiency, retaining 71% ± 3.8% of CoQ10. FTIR and DSC analyses confirmed the successful formation of the complex. The formulated serum exhibited shear‐thinning behavior, an optimal pH of 4.3 ± 0.2 closely aligning with the skin's natural acidity for enhanced compatibility—along with excellent spreadability and stability. Diffusion tests revealed a significant enhancement in solubility when CoQ10 was complexed, effectively overcoming its solubility barrier. Irritation tests validated the serum's safety for topical use.

**Conclusion:**

This study successfully developed a CoQ10 serum that overcame its solubility limitation, demonstrating favorable properties for skincare application. With its strong physicochemical characteristics and biocompatibility, this formulation shows significant promise for broader incorporation into skincare products.

**Abbreviations**
CoQ10: coenzyme Q10
DSC: differential scanning calorimetry
EE: encapsulation efficiency
FTIR: fourier transform infrared spectroscopy
HA: hyaluronic acid
HPβCD: hydroxypropyl‐β‐cyclodextrin
ICH: International Council for Harmonization
LD: loading efficiency
OECD: Organisation for Economic Co‐operation and Development
*R* ^2^
: correlation coefficient
RSD: relative standard deviation
SD: standard deviation
UV: ultraviolet

## 1 Introduction

Skin aging is a complex biological process characterized by a progressive decline in the structural and functional integrity of the skin, leading to visible signs of aging and increased susceptibility to skin‐related disorders [1]. Skin aging is thought to be the result of both intrinsic and extrinsic factors. Intrinsic factors are determined by genetics and occur naturally, while extrinsic factors are the consequences of environmental factors that can accelerate the aging process [2]. Photoaging caused by ultraviolet radiation exposure represents the most significant cause of extrinsic aging [3]. However, various factors including poor nutrition, sleep deprivation, stress, and smoking are major contributors to skin aging [4]. The primary characteristics of skin aging can be categorized into several key areas. Wrinkles and fine lines are among the most noticeable signs of aged skin. As we age, the skin's collagen and elastin fibers22 (provide support and elasticity) begin to break down leading to the formation of wrinkles and fine lines [5]. Dryness is another symptom of aging skin that happens due to decreased sebum production and a reduced ability to retain moisture, which can result in a rough or flaky texture [6]. Loss of elasticity is another feature of aging skin resulting in sagging skin [7]. Age spots and hyperpigmentation also become more prevalent with age, as sun exposure and natural aging processes can result in the overproduction of melanin, leading to the formation of age spots, freckles, and other areas of hyperpigmentation [8]. Decreased blood circulation is another factor contributing to skin aging due to reduced blood flow to the skin that can cause a pale or dull complexion, as well as slower wound healing. Moreover, subcutaneous fat loss and bone loss occur as we age leading to a hollow or sunken appearance, particularly around the eyes and cheeks [9, 10].

**FIGURE 1:**
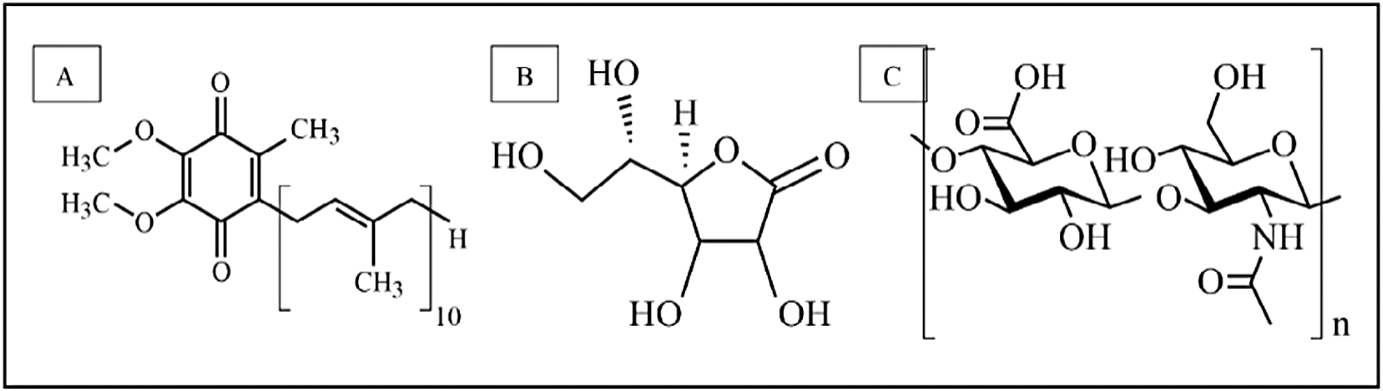
Chemical structure of (A) ubiquinone (coenzyme Q10); (B) vitamin C; (C) hyaluronic acid.

A wide range of anti‐aging products have been continuously developed to target various aspects of skin aging. Common ingredients in anti‐aging products include retinoids (vitamin A derivatives), peptides and growth factors, alpha and beta hydroxy acids, hyaluronic acid (HA), niacinamide (vitamin B3), and antioxidants, including vitamins C and E, green tea extract, and ubiquinone or Coenzyme Q10 (CoQ10) [10]. Ubiquinone (CoQ10) is a benzoquinone with a molecular weight of 863.3 g mol^−1^ and a chemical formula of C_59_H_90_O_4_ [11] (Figure 1A). CoQ10 acts as an anti‐aging agent by reducing oxidative stress. It is a potent antioxidant that acts by neutralizing free radicals and reducing oxidative stress in skin cells. By this, it helps prevent oxidative damage to cellular components like collagen and elastin, which are critical for maintaining skin structure and elasticity. It also regenerates other antioxidants, like vitamin E, enhancing the skin's ability to counteract aging [12].

CoQ10 is also essential for mitochondrial function and energy production. It enhances ATP synthesis, improving cellular metabolism and promoting cell turnover and repair. This supports the maintenance of skin cells and helps them resist aging‐related damage. Besides, it has an anti‐inflammatory and photoprotection effect, counteracting inflammation and light, the two problems that enhance skin aging [13]. All these functions help maintain a youthful appearance, reduce wrinkles, and combat signs of aging caused by environmental and intrinsic factors [11].

Vitamin C (ascorbic acid) is a crucial water‐soluble micronutrient vital for various biological processes, especially skin health. Since humans cannot synthesize vitamin C, it must be obtained through diet or supplements. One of its primary roles is in collagen synthesis, essential for skin strength, elasticity, and structure. Vitamin C facilitates the hydroxylation of proline and lysine, aiding proper collagen formation, which is vital for skin maintenance and combating aging. As a powerful antioxidant, it neutralizes free radicals, preventing cell damage, collagen breakdown, and elastin fiber degradation [14]. Additionally, vitamin C provides protection against UV radiation by neutralizing free radicals generated by sun exposure and working synergistically with vitamin E to reduce oxidative damage. Its involvement in wound healing, through collagen synthesis and immune regulation, further underscores its importance. Topical application of vitamin C in serums or creams enhances skin concentration, promoting collagen production and reducing signs of aging, making it key in skincare products [15].

The primary molecule involved in maintaining skin moisture is considered to be hyaluronan or hyaluronic acid (HA), known for its exceptional ability to bind and retain water molecules. HA not only contributes to the skin's structural integrity but also plays a crucial role in maintaining skin hydration as we age [16, 17]. HA is a long, unbranched polysaccharide composed of repeating disaccharide units of D‐glucuronic acid and N‐acetyl‐D‐glucosamine. The molecular weight of HA can vary greatly from a few thousand Daltons to several million Daltons (Figure 1C). HA can hold up to 1000 times its weight in water, contributing to its moisturizing and water‐retention properties in cosmetic and skincare formulations [18, 19]. As people age, the body's production of hyaluronic acid (HA) declines, leading to decreased skin hydration and elasticity, which contributes to wrinkles and fine lines. Additionally, the molecular weight of HA shifts to smaller, less effective fragments that reduce water retention and structural support. This decline weakens the skin's barrier, making it more vulnerable to UV radiation and external stressors, which accelerate aging [16]. To combat these effects, HA is commonly used in anti‐aging skincare products, including serums and creams, to restore hydration. HA‐based dermal fillers are also injected to temporarily restore volume and smooth out wrinkles, making HA a key ingredient in cosmetic formulations [20, 21].

The aims and objectives of this study are the solubilization of ubiquinone in water by complexation with HPβCD and incorporation of this complex in an HA serum containing vitamin C to produce a stable and effective serum for skin care application with good physical and chemical integrity.

Hydrophilic materials face challenges in penetrating the skin due to the lipophilic nature of the stratum corneum. As a result, hydrophilic compounds are absorbed less efficiently. To enhance skin absorption, penetration enhancers, specialized formulations like emulsions, or occlusive methods that hydrate and increase skin permeability are often required. In this work, the goal is to formulate hyaluronic acid, a highly moisturizing agent, which users opposed to lipophilic formulations that result in an unwanted greasy texture. By making the fat‐soluble CoQ10 water‐soluble, the hyaluronic acid will increase moisture content and improve the permeability of the water‐soluble complex. Additionally, the complex will offer a gradual release of CoQ10, preventing it from being quickly washed away by the dermis, potentially enhancing its effectiveness.

## 2 Material and Methods

### 2.1 Materials

Ubiquinone (purity 99.2%) and hydroxypropyl β‐cyclodextrin (purity 99.6%) were obtained from Wacker Chemie AG (German). Glycerol, phenoxyethanol, and propylene glycol were obtained from Fisher Scientific (USA); *Aloe vera* extract from Coats Aloe International Inc. (USA); vitamin C from Sigma Aldrich (USA, 99.5%); xanthan gum from Cargill (USA); glyceryl stearate from BASF Pharma (Germany); caprylic/capric triglyceride from IOI Oleochemicals (Malaysia), green tea extract from Naturex (France); hyaluronic acid from Bloomage Freda BioPharm (China); citric acid from Otsuka Pharmaceutical (Japan), and sodium benzoate from Cargill (USA). All other organic solvents or materials were of HPLC, analytical, or pharmaceutical grade.

### 2.2 Animals

Healthy male Wistar rats weighing 250 ± 15 g were acclimatized and housed individually at the Laboratory Animal Research Unit of the Applied Science University. The animals were kept 12‐h cycles of light and darkness with free access to water and food (Purina‐Labdiet, USA) 1 week prior to the experiment. Fasting started at the night of the experiment (12 h) with only access to water.

The study received ethical approval from the Al‐Ahliyya Amman University ethical committee (Decision no. 2/3‐2022‐2023 AUP: AAU) and from the Applied Science University.

### 2.3 Methods

#### 2.3.1 Development of Coenzyme Q10 Method of Analysis

A UV spectrophotometer was used for the detection and quantification of free CoQ10 and its complexes. Optimal solubility was achieved utilizing an 80:20 methanol to water ratio. A stock solution of CoQ10 was subsequently prepared by dissolving 50 mg of CoQ10 in 50 mL of the solvent system with gentle stirring. The solution was then filtered to yield a clear solution. Further dilution to a concentration of 50 μg mL^−1^ was prepared using the same solvent system. For the spectral analysis, this solution at 50 μg mL^−1^ concentration was scanned over a wavelength range of 200–400 nm using a UV spectrophotometer. The solvent system (methanol:water 80:20) was utilized for both the blank and the control. To ensure the specificity and accuracy of the method when measuring CoQ10 and its prepared complexes, hydroxypropyl beta cyclodextrin (HPβCD) was also scanned at 200–400 nm range.

#### 2.3.2 Validation of Analytical Methods

The analytical method employed in this study was validated in accordance with the International Council for Harmonization (ICH) guidelines. Different dilutions of CoQ10, prepared from the stock solution in methanol–water solvent, were employed for the validation tests.

The linearity of the developed method was validated. A series of CoQ10 concentrations was prepared and analyzed to derive a calibration curve. Specifically, six concentrations of CoQ10—100, 125, 250, 500, 750, and 1000 μg mL^−1^—were prepared from the stock solution, and their absorbance values were measured at 276 nm. Each concentration was measured thrice, and the average absorbance value ± standard deviation (SD) was recorded. The absorbance values were then plotted against the corresponding concentrations to create a calibration curve.

The precision and accuracy of the developed method were evaluated by analyzing a set of samples using both intra‐day (within‐run) and inter‐day (between‐run) measures. Intra‐day precision was evaluated by analyzing six individual samples (at the calibration concentrations) with three replicates each on the same day. This helped to determine the consistency of the measurements within a single analytical run. Inter‐day precision was assessed by re‐analyzing the same six samples on different days following the same procedural steps. This aimed to evaluate the consistency of the method over different runs on different days, which is crucial for maintaining reliable measurements over time. The precision of the method was quantified in terms of the relative standard deviation (RSD). According to the ICH guidelines, an acceptable coefficient of variation (CV%) should be below 2%, which was the threshold used in this study. Accuracy was established using a sample concentration of 250 μg mL^−1^ as it falls within the linearity range established by the calibration curve.

For the recovery test, a total of 0.5 g of the CoQ10 serum was dissolved in 100 mL of methanol. The solution was shaken vigorously to ensure complete solubilization of CoQ10. After stirring, the mixture was filtered to ensure the removal of any insoluble material. The filtrate was then diluted using the methanol–water solvent (80:20 ratio) for spectrophotometric analysis. These dilutions were assessed in triplicates, and the average absorbance value ± SD was recorded. The percentage of recovery was then calculated.

The specificity and selectivity of the developed analytical method were evaluated using a placebo serum. This involved scanning the placebo at the predetermined *λ*_max_ to assess if any background absorbance was observed at this wavelength, which would potentially indicate interference by substances other than CoQ10. The placebo serum was tested using serial dilutions with 80:20 methanol–water solvent system. Absorbance at the *λ*_max_ for each dilution was recorded and analyzed. This procedure enabled to determine if the developed method was specific for CoQ10. Also, a full spectral scan at 200–400 nm was performed on the placebo serum. This broad‐range scan provided a comprehensive spectral profile that could be compared with the CoQ10 spectrum to further confirm the method's selectivity.

#### 2.3.3 Preparation of Q10–HPβCD Inclusion Complexes

The HPβCD and CoQ10 inclusion complexes were prepared by two different methods: the kneading and trituration methods [22]. Each method was carried out at different HPβCD to CoQ10 molar ratios as shown in Table 1.

**TABLE 1:** Samples of Q10–HPβCD complexes prepared by kneading and trituration methods illustrating the weight ratios of the six prepared formulations and method of preparation.

Sample code	Amount of CoQ10/Sample (mg)	Amount of HPβCD (mg)
Kneading method		
S1	50	50
S2	50	100
Trituration method		
S3	50	50
S4	50	100
S5	100	50
S6	150	50

The kneading process was initiated by weighing 50 or 100 mg of HPβCD. The weighed HPβCD was placed into a mortar and mixed with enough water to form a thick paste. Gradually, 50 mg CoQ10 was incorporated into this paste. This resulted in HPβCD to CoQ10 molar ratios of 1:1 or 1:2. This mixture was then kneaded manually, maintaining a consistent direction, for a duration of 1 h. This step ensured the homogenous incorporation of CoQ10 into the HPβCD paste. Following the kneading process, an additional amount of water was added to maintain the paste consistency. The paste was then left to dry at a controlled temperature between 37°C and 40°C for a period of 2–4 h. Once the drying phase was completed, the dried mixture was gently crushed using a mortar and pestle to obtain a fine powder. The crushed mixture was then sieved through a mesh sieve (#65) to ensure a uniform particle size. The produced inclusion complex was stored in a sealed container to prevent contamination or degradation.

In the trituration method, HPβCD and CoQ10 at predetermined weights according to the desired ratios (1:1, 1:2, 2:1, and 3:1) were combined in a mortar. The components were then thoroughly triturated for a duration of 1 h to ensure proper blending and formation of the inclusion complex. As this method did not involve any solvent, there was no need for a drying step. After trituration, the mixture was directly collected. The samples were stored in a desiccator to maintain dryness and prevent exposure to atmospheric moisture prior to further analysis. Samples codes and ratios are listed in Table 1.

### 2.4 Characterization of the Prepared Complexes

#### 2.4.1 Fourier Transform Infrared Spectroscopy (FTIR)

FTIR spectra were carried out with FTIR (Shimadzu, Japan) using a potassium bromide disk method and scanned from 4500 to 500 cm^−1^. They were utilized as a mean to confirm the formation of the CoQ10–HPβCD complexes and to analyze their structural properties. The spectra for pure CoQ10 and HPβCD were also obtained, and all spectra were recorded.

#### 2.4.2 Differential Scanning Calorimetry (DSC)

The DSC was carried out as a supplementary method to provide further evidence of complex formation. CoQ10, HPβCD, and the prepared CoQ10–HPβCD complexes were scanned in a controlled temperature range from 4°C to 300°C at a heating rate of 10°C min^−1^. The resulting thermograms were then carefully studied.

#### 2.4.3 Solubility of the Complex in Water

The water solubility of the prepared CoQ10–HPβCD complexes was assessed. Six test tubes were prepared, each corresponding to one of the six prepared samples. The first six test tubes were labeled S1 through S6, and each received 1 mL of distilled water, followed by an excess amount of the respective CoQ10–HPβCD complex. A seventh test tube was needed that contained 1 mL of distilled water and pure CoQ10. This last tube served as a control to demonstrate the inherent water solubility of CoQ10 without any complexation.

Once the samples were prepared, they were placed in a controlled environment maintained at 25°C. The test tubes were then shaken at a speed of 50 rpm for a duration of 24 h. This process facilitated the attainment of equilibrium between the soluble and insoluble forms of the complexes in each test tube. After the completion of the 24‐h period, each solution was carefully filtered. The filtrates were then ready for further analysis to quantify the amount of CoQ10 dissolved in water, which represented the solubility of each complex.

#### 2.4.4 Determination of Drug Content

The quantification of CoQ10 content in the prepared complexes was critical to assess the efficiency of the complexation process and the efficacy of the method used. This procedure was based on the results obtained from the solubility study. For each sample, an equivalent volume of the soluble fraction was added to 10 mL of methanol, which served to extract the soluble CoQ10 from the complex. To ensure complete extraction, the mixture was stirred continuously for 1, 2, and 3 h. These time intervals were investigated to optimize the extraction process. Following each extraction period, the samples were filtered. The filtrate was then diluted appropriately, and its absorbance was read three times at 276 nm. The average of these readings was taken, and the corresponding concentration of CoQ10 was determined.

#### 2.4.5 Stability of the Dry Complex

The stability of the prepared CoQ10–HPβCD complex was evaluated approximately over a period of 4 months. This assessment aimed to monitor potential changes in the properties of the complex over time, which is critical for the practical application and storage of the formulated complexes. For this purpose, 1 g of the selected complex (chosen based on its superior solubility and drug loading characteristics) was preserved. The complex was stored in an Eppendorf tube, which was then wrapped in aluminum foil to minimize exposure to light, a factor known to influence CoQ10 stability. The sample was then left at ambient laboratory conditions. At each monthly interval, a 100 mg portion of the stored complex was taken for analysis. The analysis involved re‐evaluating the content of CoQ10 and the water solubility of the complex. This process has provided an accurate overview of any changes in the physicochemical characteristics of the complex that might have occurred and helped to establish its stability profile over the storage period.

### 2.5 Preparation of the Serum

The formulation of all serum was performed as illustrated in Table 2. An amount of 200 g of the serum was prepared using CoQ10 complex. For comparison purposes, a control formulation was also prepared using free CoQ10.

**TABLE 2:** The composition of the serum formula showing the percentage of each ingredient and the function of each one in the final serum formula.

Phase no.	Ingredients	Master formula % (w/w)	Function
A	Deionized water	Qs 100	Vehicle
	Glycerol	7	Humectant and emollient
	Propylene glycol	3	Humectant
	Xanthan gum	0.2	Viscosity modifier
	Disodium EDTA	0.1	Chelating agent
B	Glyceryl stearate	3	An emulsifier, stabilizing ingredient
	Caprylic/capric triglyceride	4	An emollient, dispersing agent, and solvent
C	Green tea extract	1	Antioxidant activity, de‐pigmenting effect
	Hyaluronic acid	1	Conditioning agent
	*Aloe vera* extract	1	Skin conditioning agent, moisturizing
	**Q10 complex**	**Complex equivalent to 2%**	**The API**
	Citric acid	0.2	Buffering agent
	Vitamin C	0.5	Antioxidant
	Sodium benzoate	0.4	Preservative
	Phenoxyethanol	1	Preservative
	Perfume (lavender)	0.2	Flavoring agent

The preparation process of each serum was carried out in a systematic and controlled manner, following a three‐phase method. This approach involved the initial preparation of two distinct phases (Phase A and B), which were then combined to form an emulsion before a final Phase C was added. In Phase A, all the ingredients necessary for the formulation were added to water in a specified container. This mixture was then heated to a temperature of 70°C while being continuously stirred. Phase B was simultaneously prepared in a separate container. This involved mixing all the designated Phase B ingredients and heating this mixture to the same temperature of 70°C. To ensure thorough mixing and heating, agitation was maintained throughout this process. Once both Phase A and Phase B were adequately produced and uniformly heated, they were combined by adding Phase B to Phase A while maintaining continuous stirring to ensure complete emulsion formation. Following that, the temperature of the mixture was allowed to cool down to room temperature. At this point, the ingredients of Phase C were successively added, and the prepared sera were ready for further analysis.

### 2.6 Evaluation of the Prepared Serum

Following the preparation of the sera, a rigorous evaluation was conducted to assess the quality and characteristics of each serum.

#### 2.6.1 Determination of CoQ10 Content

The content of CoQ10 in the serum was determined through an extraction method coupled with UV spectrophotometric analysis. Specifically, a sample of 1 g of the serum was placed into a conical flask, to which 10 mL of methanol was added. The mixture was then shaken vigorously, and an additional 10 mL of methanol was introduced, and the flask was placed on a magnetic stirrer for 1 h. This allowed for complete extraction of the CoQ10 present in the serum sample. After the stirring process, the mixture was filtered twice to remove any undissolved material. The filtered solution was then properly diluted, and the absorbance was subsequently measured three times at 276 nm. The mean value of these measurements was used to determine the content of CoQ10 in the serum.

#### 2.6.2 Viscosity

A Physica MCR 302 rheometer (Anton Paar) was employed to study the rheological behavior of the prepared serum. All measurements were carried out at a temperature of 25°C ± 1°C, using spindle Cp 50. A sample volume of 5–10 mL of the serum was loaded between the concentric cylinders of the rheometer. This volume ensured the accuracy of the measurements. The rheological behavior of the serum was analyzed by constructing flow curves. These were produced by plotting viscosity against shear rate, providing insights into the flow properties of the serum under varying shear rates. In addition to the flow curves, viscosity–temperature curves were also plotted.

#### 2.6.3 pH of the Serum

The pH of a skincare product markedly influences its compatibility with the skin and its stability. Human skin has a slightly acidic pH [23]. To ensure optimal compatibility with the skin and to minimize any potential irritation, it is recommended that the pH of topical skincare products also be slightly acidic. For pH measurement, a small sample of the serum was placed in a clean and dry beaker. The electrode of the calibrated pH meter was carefully dipped into the sample, ensuring it was fully submerged but not touching the bottom of the beaker. The pH reading was then recorded.

#### 2.6.4 Spreadability

This test was performed according to Sabale, Kunjwani and Sabale [24]. In this experiment, a fixed weight of 1 g of the prepared serum was spread onto a premarked circle with a diameter of 2 cm on a glass plate. A second glass plate was then carefully placed on top of the spread serum. To apply the required pressure, a half kilogram weight (obtained by using a beaker filled with water to the target weight) was placed on the top glass plate and left for 5 min. This method mimics the pressure typically applied when a user applies a product to their skin. After the elapsed time, the weight was removed, and the new diameter of the serum spread was measured. The spreadability of the serum is directly proportional to the increased diameter, indicating how well the serum can spread under a given amount of pressure. This measurement was taken three times for consistency, and the average was determined.

#### 2.6.5 Dilution Test

The serum sample containing the CoQ10–HPβCD complex was initially prepared, and 1 g of the serum was carefully weighed using an analytical balance. The serum sample was transferred to a clean, dry beaker, and 20 mL of deionized water was added to ensure that the serum sample was fully submerged. The mixture was continuously stirred at a constant speed of 400 rpm using a magnetic stirrer until a homogenous solution was achieved [25]. The solution's volume was then gradually increased; 10 mL aliquots of deionized water were added to the beaker at regular intervals. After each addition, the solution was stirred for a further 10 min to ensure thorough mixing. The dilution process was continued until the total volume of solution in the beaker reached 100 mL. Following each dilution step, the solution was observed for any visible signs of phase separation, sedimentation, or precipitation.

#### 2.6.6 Drug Release and Diffusion

To predict the efficacy of the prepared serum, the formulation was subjected to an in vitro diffusion study, which aims to emulate the product's performance upon application onto human skin. For this purpose, a Franz Diffusion Cell model was used. The receiver compartment of the diffusion cell was filled with 12 mL of the serum. The diffusion media consisted of a phosphate buffer solution at pH 7.0. The area of diffusion was established at 1.7 cm^2^. Two sets of samples were prepared for this experiment. For the first set, 200 mg of the CoQ10 serum was added to each of three cells. For the second set, the control formula was added to each of three other cells. Readings were taken at intervals of 0.5, 1, 2, 3, 4, 5, 6, 7, 8, and 24 h. To maintain the sink conditions, the media was replaced with fresh buffer after each reading. Due to the light sensitivity of CoQ10, special precautions were taken during the experiment to prevent light exposure. The entire diffusion apparatus was covered with foil, and the laboratory lights were turned off, and the curtains were closed to provide an optimal environment for the experiment.

### 2.7 Irritation Test

The irritation test was conducted on two healthy male Wistar rats, each weighing approximately 250 ± 15 g. Each rat was assigned a different treatment; rat number 1 was used as a negative control and received no treatment, and rat number 2 was treated with the CoQ10 serum formula. The rats were maintained under controlled conditions at a temperature of 20°C ± 3°C, a humidity of 50% ± 15%, and a photoperiod cycle of 12 light/12 h dark. They were provided with a conventional laboratory diet and an unrestricted supply of drinking water [26, 27].

Prior to the experiment, the dorsal area of each rat's trunk was closely clipped to remove fur, ensuring humane restraint of the rat during the process. Once the healthy and intact skin was ensured, the rats were prepared for the application of the CoQ10 serum formulation. The formula was applied over an area of approximately 6 cm^2^ of skin and covered with a gauze patch.

According to the OECD (Organisation for Economic Co‐operation and Development) guideline, an initial test was conducted by applying one patch and removing it after 4 min. If no serious skin reaction was observed, a second patch was applied and removed after 1 h. Thereafter, third, fourth, and fifth patches were applied and left for 4, 12, and 24 h, respectively. The animals were examined immediately after patch removal for signs of erythema and edema, and dermal reactions were scored. The assessment of dermal irritation was conducted following Draize's dermal irritation scoring model as per OECD recommendations (Table 3) [28].

**TABLE 3:** Draize dermal irritation scoring system showing the scale of erythema and edema (26).

Erythema and eschar formation	Value	Edema formation	Value
No erythema	0	No edema	0
Very slight erythema (barely perceptible)	1	Very slight edema (barely perceptible)	1
Well‐defined erythema	2	Slight edema (edges of area well defined by definite raising)	2
Moderate‐to‐severe erythema	3	Moderate edema (raised approximately 1 mm)	3

### 2.8 Stability Study

The stability of the final serum product was evaluated over a 3‐month period. The primary focus of this study was on the physical examination of the product and the assessment of CoQ10 content within the serum. The study was conducted at room temperature, which was controlled within the lab environment during the day at 24°C–26°C. To mitigate any potential effects of light exposure on the stability of the product, the containers holding the serum samples were carefully wrapped in aluminum foil.

On a monthly basis, three serum samples were taken for testing in addition to the initial time zero collection. Alongside the physical examinations, the CoQ10 content in the serum was determined and recorded. This determination was conducted thrice for each sample, and an average of these readings was taken to ensure accuracy and reliability of results. The evaluation of CoQ10 content at each time point offered valuable insights into the stability of the serum over the study period.

## 3 Results and Discussion

### 3.1 Method Development

**FIGURE 2:**
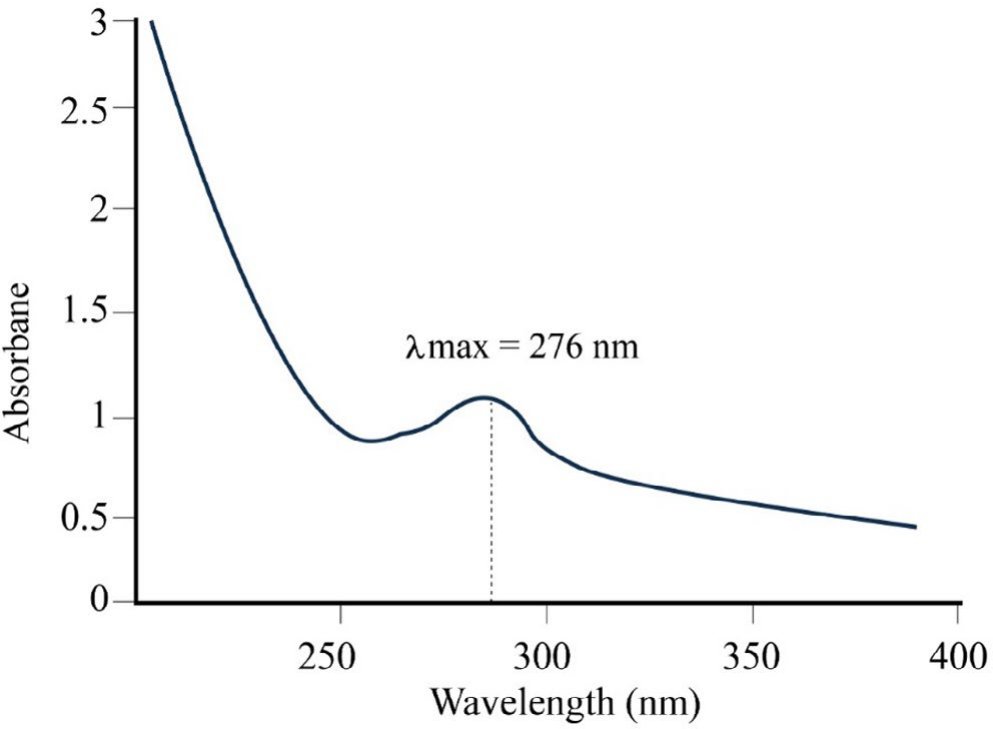
UV scan of CoQ10 showing the lambda max at 276 nm.

To determine the optimal wavelength (*λ*_max_) for the absorption of Q10, a sample solution was subjected to UV–Vis spectroscopic analysis. The tested solution had a concentration of 50 μg mL^−1^ CoQ10 in a solvent mixture of methanol:water at a ratio of 80:20. The scanning results as illustrated in Figure 2 revealed a distinct peak with a maximum absorbance at a wavelength of 276 nm. This wavelength represents the point of maximum absorption for CoQ10 in the given solvent mixture. These findings are consistent with previous literature. El‐Leithy and Abdel‐Rashid [29] reported that CoQ10 exhibits *λ*_max_ at 276 nm in methanolic solutions. Similarly, Wu et al. [30] conveyed that in a methanol‐n‐hexane (85:15) solvent system, the *λ*_max_ of CoQ10 was also located at 276 nm. Also, Orozco et al. [31] utilized the 276 nm for the quantification of CoQ10 in dietary supplements.

#### 3.1.1 Method Validation

**FIGURE 3:**
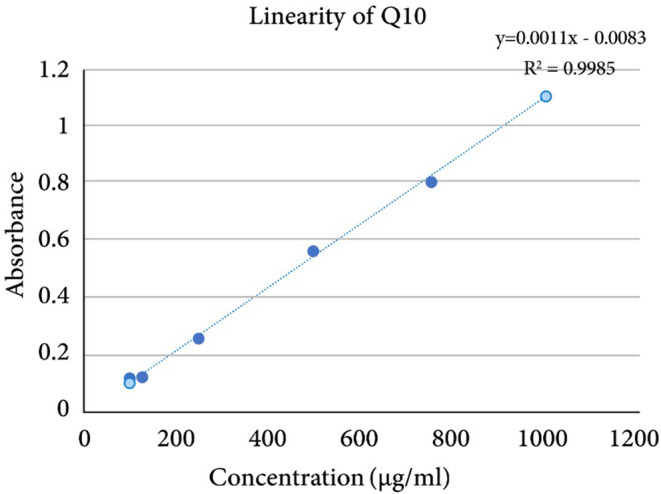
The linearity plot of Q10 showing the linear regression equation and correlation coefficient.

To ascertain the linearity of the absorbance–concentration relationship for CoQ10 in the methanol:water (80:20) solvent system, a series of solutions with varying concentrations were prepared and analyzed. For a robust linear correlation, a minimum of six concentration points is recommended. Our study encompassed a concentration range of 100–1000 μg mL^−1^ for which the absorbance at 276 nm was measured and a linear relationship was established. The correlation coefficient (*R* ^2^) for the linearity test was computed to be 0.9985, indicating an exceptionally strong linear relationship between the absorbance and the concentration of CoQ10 within the tested range. A correlation coefficient nearing 1 is indicative of a near‐perfect linear correlation, and the observed value confirms the appropriateness of the UV–Vis spectroscopic method for quantifying CoQ10 in the specified concentration range. The graphical representation of this linear relationship of concentration against absorbance is shown in Figure 3.

For all subsequent assays pertaining to the quantification of CoQ10 in this study, it was imperative that the dilutions produce concentrations within the 100–1000 μg mL^−1^ range. This approach ensured that the readings obtained were linearly related to the concentrations, thereby guaranteeing the precision and accuracy of the measurements. The established linearity within the 100–1000 μg mL^−1^ range did validate the UV–Vis spectroscopic method used and also reinforced the importance of maintaining sample concentrations within this range for reliable and consistent readings.

All measurements were conducted over two consecutive days to assess both inter‐day and intra‐day precision for CoQ10 quantification. The computed RSD values for all measurements ranged between 1.6 and 1.68, aligning with the acceptable threshold prescribed by the ICH guidelines. As per ICH guidelines, an RSD not exceeding 2.0 is regarded as indicative of acceptable precision. Furthermore, the accuracy or recovery rate of CoQ10 from the samples spanned between 95.4% and 102.3%. Notably, the ICH guideline stipulates an acceptable accuracy range of 90%–110%. To ascertain the specificity and selectivity of our method for CoQ10 determination at 276 nm, a placebo serum, devoid of any CoQ10, was analyzed under similar conditions. The placebo serum at 1:10 dilution ratio with methanol:water (80:20) was analyzed, and the recorded absorbance was around 0.25 ± 0.02. This suggests the presence of some constituents in the placebo serum that may absorb light in a proximity to the *λ*_max_ of CoQ10. However, further diluting the placebo serum to a ratio of 1:20 and greater with the same solvent system produced negligible absorbance of values below 0.01. Given these findings, all samples for accurate CoQ10 quantification should be diluted with the solvent at a ratio of 1:20 or greater. This ensures that our method remains selective for CoQ10 and is uninfluenced by any other endogenous serum components that may possess an absorbance close to 276 nm.

To verify the ability of the quantification method to accurately retrieve the CoQ10 from the sample matrix, a recovery study was then carried out. An initial quantity of 0.5 g of serum which theoretically contained 10 mg of CoQ10 was dissolved in 20 mL of methanol. This solution was subsequently diluted at a 1:1 ratio, and its absorbance was gauged three times to ensure consistency. The absorbance of the filtered extract was also determined, and interestingly, when subjected to a higher dilution ratio of 1:40, the results appeared to be more reliable. The rationale behind this observation could be attributed to the effect of dilution on the excipients. With increased dilution, the impact of these excipients on the overall absorbance decreases, facilitating a clearer and more accurate quantification of CoQ10. Furthermore, when a sample containing 1 g of the serum was dissolved in 20 mL of methanol, an unexpectedly high average recovery of approximately 120% was noted. This higher recovery was attributed to the added absorbance conferred by the excipients present in the serum. Considering these observations, it becomes evident that to minimize the confounding effects of the excipients and to ensure accurate recovery of CoQ10, all serum samples should be subjected to a dilution ratio exceeding 1:20.

### 3.2 Characterization of the Prepared Complex

#### 3.2.1 Fourier Transform Infrared Spectroscopy (FTIR)

**FIGURE 4:**
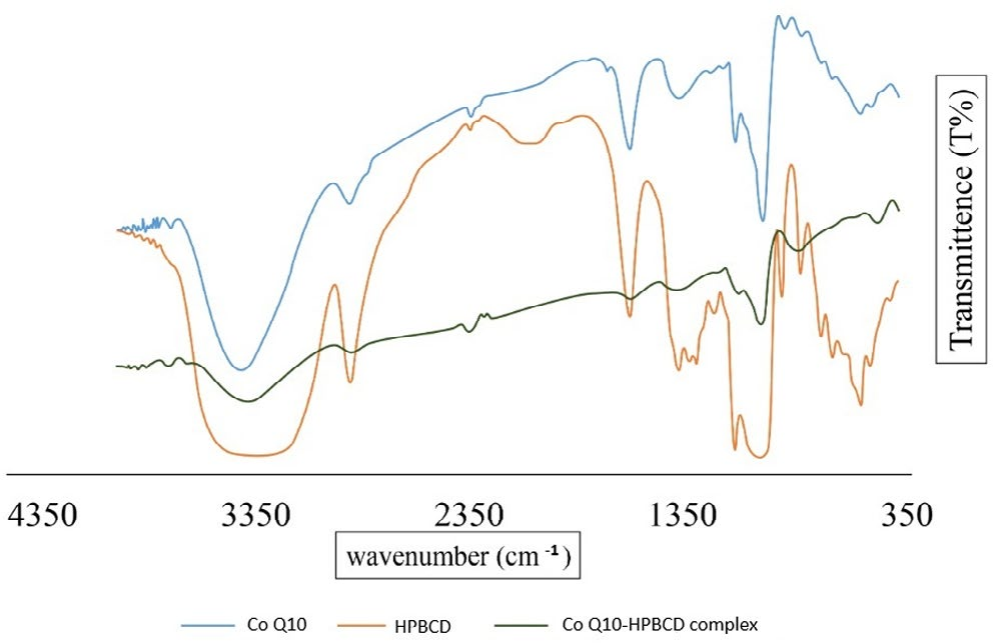
FTIR spectrum of pure CoQ10 showing the distinctive peaks at 3412.08 cm^−1^ (O‐H stretching), 1020 cm^−1^ (C‐O stretching), and 1715 cm^−1^ (carbonyl stretching); HPβCD showing distinctive peaks at 3385 cm^−1^ (O‐H stretching), 1030 cm^−1^ (C‐O stretching); and CoQ10–HPβCD complex showing hidden and slight shifting of the peaks of CoQ10 as an indication of physical interaction.

FTIR serves as a primary analytical tool to identify functional groups and detect potential interactions between molecules [32]. FTIR was employed to explore the molecular interactions within the CoQ10–HPβCD complex using spectra of pure CoQ10 and HPβCD as reference baselines (Figure 4). The FTIR spectrum of pure CoQ10 exhibited characteristic peaks at 3412.08 cm^−1^ corresponding to O‐H stretching, given that CoQ10 has a quinone structure, and 2924 cm^−1^ which may be attributed to C‐H stretching that is typical for aliphatic hydrocarbons. In the context of CoQ10, it likely corresponds to the isoprenoid side chain of the molecule and a peak of 1020 cm^−1^, which is consistent with C‐O stretching. The peak at 1715 cm^−1^ was thought to refer to the carbonyl group stretching.

The FTIR analysis of HPβCD revealed distinct peaks at 3385 cm^−1^ which is indicative of O‐H stretching, indicating the presence of hydroxyl (‐OH) groups, that is consistent with the structure of cyclodextrins which contains multiple hydroxyl groups. Also, a strong peak at 1030 cm^−1^ was observed, which could potentially be attributed to the C‐O stretching: a key feature in cyclodextrins due to their glucose subunits. These spectral features are consistent with the molecular structure and functionalities of cyclodextrins.

The FTIR spectrum of the CoQ10–HPβCD complex showed peaks at 3399, 2922.16, and 1026.13 cm^−1^ that notably showed slight deviation compared to the individual components. The peak initially observed at 3412.08 cm^−1^ in pure CoQ10 and 3385.07 cm^−1^ in HPβCD shifted to 3399 cm^−1^ in the complex. Similarly, the peaks at 2924 cm^−1^ (CoQ10) and 1020 cm^−1^ (CoQ10) shifted to 2922.16 and 1026.13 cm^−1^, respectively. The peak of the carbonyl group was almost hidden in the complex spectrum. Such spectral shifts have been documented in literature as evidence of the formation of inclusion complexes [33]. The interactions might be due to hydrogen bonding, van der Waals forces, or other intermolecular forces leading to the complex formation. The shifts in the O‐H and C‐O stretching vibrations in the complex indicate a potential interaction between the hydroxyl groups of HPβCD and CoQ10.

#### 3.2.2 Differential Scanning Calorimetry (DSC)

**FIGURE 5:**
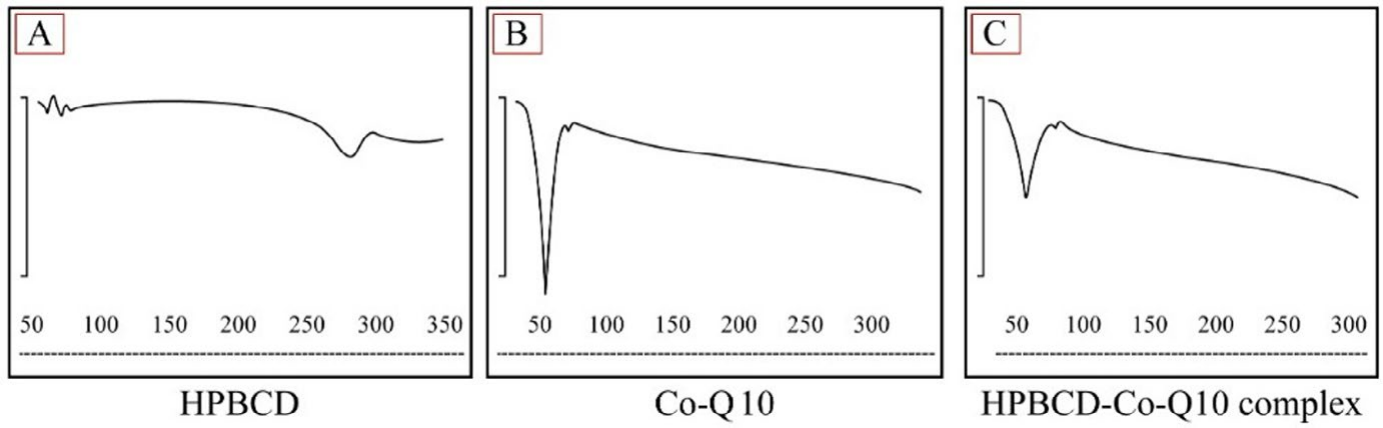
DSC analysis of the HPβCD (A) showing a wide small peak around 260°C representing melting and decomposition, CoQ10 (B) with a sharp clear peak at 55°C representing the melting point of the crystalline form, and CoQ10–HPβCD complex (C) showing a small shoetened wide peak around 50^o^C of CoQ10 melting which might indicate a physical interaction with the cyclodextrin.

DSC serves as a pivotal analytical technique for investigating thermal properties of materials, including phase transitions. In this study, DSC was employed to characterize the CoQ10–HPβCD complex, CoQ10 and HPβCD. The DSC thermogram for HPβCD demonstrated a wide endothermic event around 60°C, signifying the dehydration of the compound. Another transition was evident at 260°C, suggesting melting or decomposition of HPβCD has occurred (Figure 5A). On the other hand, the DSC thermogram of pure CoQ10 exhibited a sharp endothermic peak at 55°C, which corresponds to its melting point (Figure 5B). This prominent peak was associated with the crystalline nature of CoQ10, and this result agreed with literature values for its melting point.

In contrast to the individual thermograms of CoQ10 and HPβCD, the DSC trace of the CoQ10–HPβCD complex presented noticeable differences (Figure 5C). The endothermic peak corresponding to the melting of CoQ10 was conspicuously diminished in intensity. This distinct thermal events associated with the pure compound such as its melting can be decreased or even obliterated. This is mainly due to the encapsulation or inclusion of the molecule within the cyclodextrin's cavity hindering its regular crystalline structure. In addition, the peak was slightly shifted to higher temperature indicating the successful interaction of CoQ10 with HPβCD which led to a possible amorphous form or a changed crystalline state.

#### 3.2.3 Solubility of the Complex

The solubility of CoQ10–HPβCD complexes in water was assessed at different weight ratios of CoQ10 and HPβCD. These complexes were prepared using both kneading and triturating methods to determine the optimal formulation and procedure that provides maximum solubility.

The various weight ratios investigated were 1:1, 1:2, 2:1, and 3:1 of CoQ10 to HPβCD. The results revealed significant differences in the solubility profiles of the complexes depending on the preparation method and the specific weight ratio used. The sample that was prepared by the trituration method (2:1 (CoQ10:HPβCD) weight ratio), coded as (S5) exhibited the highest solubility of 17.5% ± 1.8%. Interestingly, for every given ratio, formulations prepared by the trituration method have consistently demonstrated enhanced solubility compared to those produced by the kneading method. The complexes formulated through the kneading method showed relatively lower solubility values. Specifically, the 1:1 and 1:2 weight ratios achieved solubilities of 4.5% and 6.3%, respectively.

Using analysis of variance (ANOVA), S1 (kneading method, 1:1 ratio) gave the least solubility. Compared to this formula, all samples gave higher solubility values in water. S2 gave nonsignificant increase in solubility (*p* = 0.1167, > 0.05), and this means the kneading method produced almost close solubility and the ratio did not affect the solubility. This is presumably due to the weak binding forces during complexation process. S3 and S5 gave highly significant increase in solubility with *p* < 0.0001 with no significant difference between them (*p* = 0.6891, > 0.05), which means this method was more efficient in the complex formation. Increasing the weight ratio further to 3:1 gave a nonsignificant decrease in solubility with S5 (*p* = 0.0819; i.e., > 0.05). To conclude, the trituration method was more efficient in increasing the solubility of Q10 using HPβCD.

#### 3.2.4 CoQ10 Content in the Prepared Complexes (EE)

Various ratios of CoQ10 and HPβCD were prepared to determine the optimal formulation regarding the encapsulation efficiency (EE). These weight ratios were 1:1, 1:2, 2:1, and 3:1 and formulated using both kneading and triturating methods. The formulation with the 2:1 ratio (CoQ10:HPβCD) (S5 formulation) prepared by the trituration method revealed the most promising encapsulation efficiency of 71% ± 3.8% with significantly higher than other ratios (*p* < 0.05). This superior EE was the ideal candidate for subsequent serum formulation, considering both its EE and its solubility in the aqueous phase. To achieve 2 g of CoQ10 in every 100 g of serum, an amount of 2.85 g of this formulation (termed as S5) is required. An overarching observation was that all formulations prepared via the trituration method have significantly outperformed those prepared by the kneading method in terms of encapsulation efficiency. The loading efficiency (LE) was also measured for all samples and was found generally higher in the trituration method than in kneading method. LE reflects the ability of the procedure to minimize loss of API during preparation. Results revealed that trituration (2:1 and 3:1) could utilize higher load of CoQ10 in respect to the total weight used. Using ANOVA, trituration method gave higher EE. Although EE of S1 and S2 was significant (*p* = 0.0257) but both are less than that obtained by the trituration method. S5 gave a significantly higher EE than S4 but not higher EE than S3 and S6 (*p* = 0.0532 and 0.0601, respectively). Based on both solubility and EE results, S5 was chosen to complete the study.

### 3.3 Characterization of the CoQ10–HPβCD Serum

#### 3.3.1 CoQ10 Content

To determine the actual amount of CoQ10 encapsulated within the CoQ10–HPβCD complex present in the serum, the previously described UV‐spectrophotometric method was employed. Samples from the serum were appropriately diluted in methanol, filtered, and analyzed for their absorbance at the *λ*_max_ of 276 nm. To determine the CoQ10 content within the serum samples, the results were interpolated with a pre‐established calibration curve with known concentrations of CoQ10. Based on this quantification method, the CoQ10 content within the CoQ10–HPβCD serum was found to be 980 μg mL^−1^, which corresponds to a recovery value of 98% ± 1.1%. This value was in line with the expected concentration, reaffirming the reliability and accuracy of the encapsulation process.

#### 3.3.2 Viscosity

**FIGURE 6:**
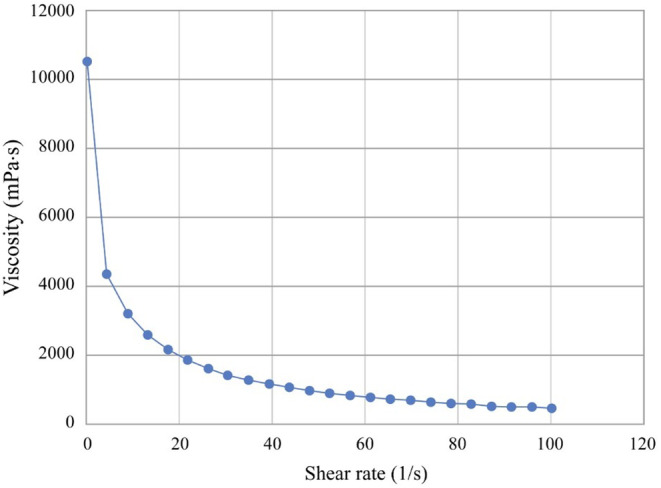
Shear‐dependent viscosity profile of CoQ10 serum showing shear‐thinning behavior, as the shear rate increases, the viscosity decreases.

**FIGURE 7:**
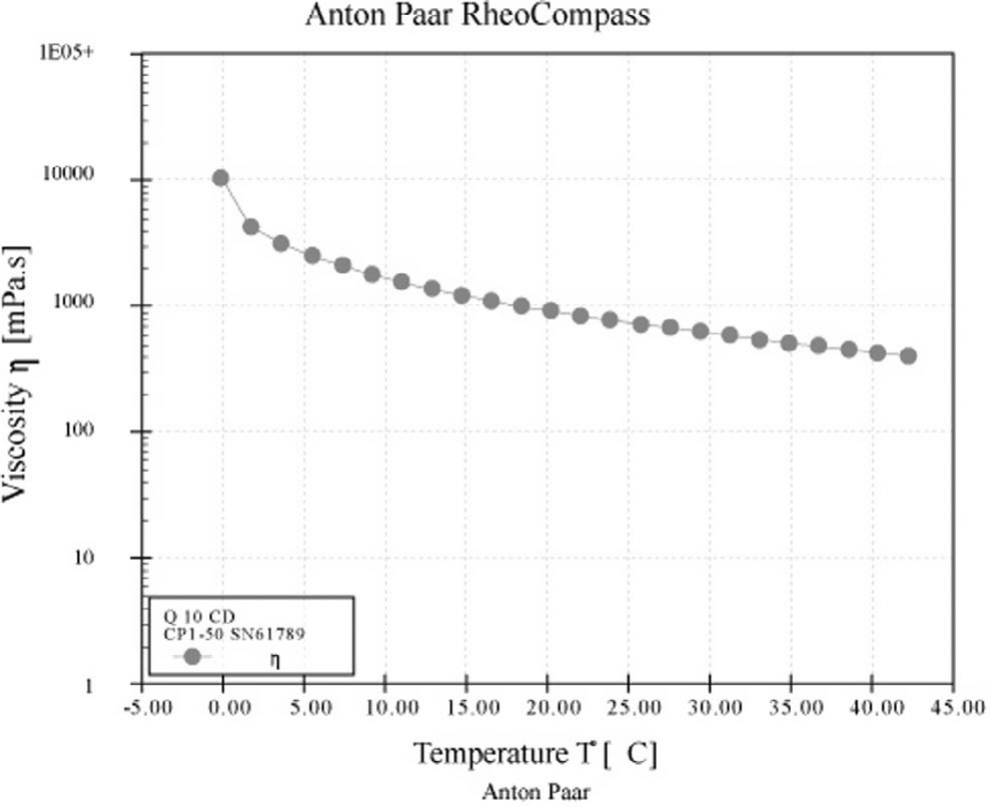
Thermo‐rheological behavior of CoQ10 serum, showing a decrease in viscosity with an increase in temperature with a viscosity value of 800 mPa s^**−1**^.

To evaluate the rheological properties of the CoQ10–HPβCD serum, measurements were performed using a rheometer. Upon investigating the rheological properties of the CoQ10–HPβCD serum, distinct variations in its viscosity were observed. This consistent decline in viscosity was observed across the entire spectrum of applied shear rates, ranging from 0.0102 to 100 s^−1^. As the shear rate increased, the viscosity generally decreased, indicating at shear‐thinning behavior typical of many complex fluids (Figure 6). This behavior where fluids become thinner or less viscous as they are stirred or subjected to shear is typical of many complex fluids including gels, creams, and suspensions. The decrease in viscosity with increasing temperature observed in the CoQ10 serum during the heating is consistent with general physical principles and observations in numerous systems [34] (Figure 7). The rationale behind this is the increased kinetic energy of molecules at higher temperatures, which leads to their enhanced movement and reduced resistance to flow. However, the decrease in viscosity from 2000 mPa s^−1^ at 5°C to 600 mPa s^−1^ at 40°C is not considered a wide range, which means the serum is able to preserve its consistency at range of temperature between refrigerator temperature and hot climate room temperature. The viscosity value of the serum was recorded as 800 mPa s^−1^ (800 cP) at 25°C, which lies within the accepted range of viscosity of topical lotion—low viscosity and easy spreadable (100–1000 cP)—as stated by the ICH (Q8). This means it is easy spreadable on the skin without any friction.

#### 3.3.3 Determination of the pH of CoQ10 Serum

The pH value plays a pivotal role in determining the compatibility and efficacy of skincare formulations. The pH of the CoQ10 serum was determined to be 4.3 ± 0.2. Healthy skin maintains a slightly acidic pH, often referred to as the “acid mantle” which lies typically between 4.5 and 5.5. This acidic environment supports barrier function, maintains hydration, and wards off potential pathogens. The pH of 4.3 for the CoQ10 serum aligns well with the natural skin pH, suggesting that the serum can potentially offer optimal compatibility and minimize skin irritation. Furthermore, a product that mirrors the skin's natural pH aids in maintaining the skin's microbiota and overall health.

#### 3.3.4 Spreadability

Spreadability is a critical parameter for topical products as it affects the product's ease of application and consumer satisfaction. It is the extent to which a product can spread over a surface area under a certain amount of pressure.

The spreadability of skincare products can markedly influence their effectiveness, coverage, and overall user experience. For the CoQ10 serum, understanding its spreadability provides insights into its potential performance when applied onto the skin. The CoQ10 serum was initially applied in a compact circle with a radius of 1 cm. Upon spreading, the serum expanded uniformly to cover a broader area, reaching a circle with a radius of 5 cm and a circumference of 31.42 cm. This represents a significant increase in the spread area, indicating that the serum spreads effortlessly and covers a considerably larger skin area from a relatively small initial area. This efficient spreadability suggested that users would not require excessive amounts of the serum for optimal coverage that leads to an economically used product. The serum's ability to evenly spread without accumulating ensures a uniform distribution crucial for even absorption of its active ingredients and the overall efficacy of the product [35]. Thus, the CoQ10 serum's uniform spreadability ensures a nongreasy and pleasant skin feel.

#### 3.3.5 Dilution Test

In the effort to assess the stability and consistency of the CoQ10 serum when subjected to dilution, a series of dilution tests were performed. The results unequivocally demonstrated that the serum's resilience and stability upon dilution. CoQ10 serum did not exhibit any indications of change in its physical or chemical properties. Importantly, there was no evidence of breaking, separation, or the disintegration of serum components regardless of the dilution level. This steadfastness suggested a well‐formulated and stable product, which can remain consistent even when exposed to variations in its solvent volume.

#### 3.3.6 Diffusion Study

**FIGURE 8:**
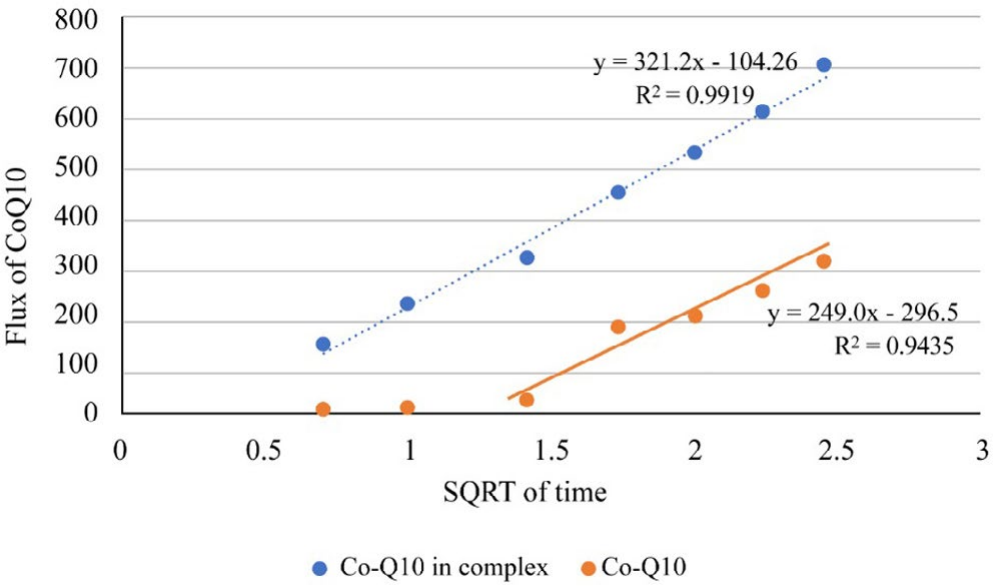
Diffusion plot of serum contained CoQ10 as HPβCD complex and the control formula contained CoQ10 without complex.

The diffusion study (Figure 8) was designed to investigate the release behavior of CoQ10 from two different formulations: a complex formula containing CoQ10 as an HPβCD complex and a control formula with CoQ10 in the absence of any complex. Initial observation of the test showed that after 0.5 h, the complex formula released 12.1% ± 1.5% with a flux of 141.17 μg cm^−2^. On the contrary, the control formula demonstrated a noticeably lower release of 0.99% ± 0.01% with a flux of 11.70 μg cm^−2^. As time progressed, the release from the complex formula consistently outperformed the control. At the 6‐h time point, the complex formula released 60.65% ± 3.0% and a flux of 705.88 μg cm^−2^, whereas the control formula lagged with a release of 26.35% ± 2.0% and a flux of 310.22 μg cm^−2^. At the end of the test period of 24 h, the complex formula had reached a release of 68.47%, while the control formula achieved 41.01%.

It seems that the complex formula has consistently showed higher flux values across all time intervals, indicating a more efficient diffusion rate. To compare the diffusion rates, the complex formula recorded a rate of 321 μg cm^−2^, whereas the control formula registered 249 μg cm^−2^. In fact, the HPβCD complex formulation of CoQ10 manifested superior performance in terms of both the volume of release and the diffusion rate over the 24‐h observation period, indicating the potential advantage of the complex formulation to produce a more regulated and prolonged delivery of CoQ10. The release kinetics of a drug or active ingredient is closely tied to the carrier system used in the formulation. The disparity in the lag times between the two formulations could be attributed to the intrinsic properties of the carrier system in the complex formula, which appears to have facilitated a rapid release of CoQ10. This release exhibited by the complex formula could particularly be beneficial in scenarios where an immediate pharmacological effect is desired. Furthermore, the overall higher release and flux from the complex formula across the observational period indicate enhanced diffusion characteristics. The percent cumulative release of CoQ10 after 6 h was statistically higher from the complex formula (60.65 ± 3.0 μg cm^−2^) compared to the control formula (26.35 ± 2.0 μg cm^−2^) (*p* < 0.0001). The same result was obtained by comparing the flux (705.88 ± 10.6) from complex formula compared to the control formula which gave (310.22 ± 8.9) (*p* < 0.0001)as shown in Figure 8.

### 3.4 Irritation Study

**FIGURE 9:**
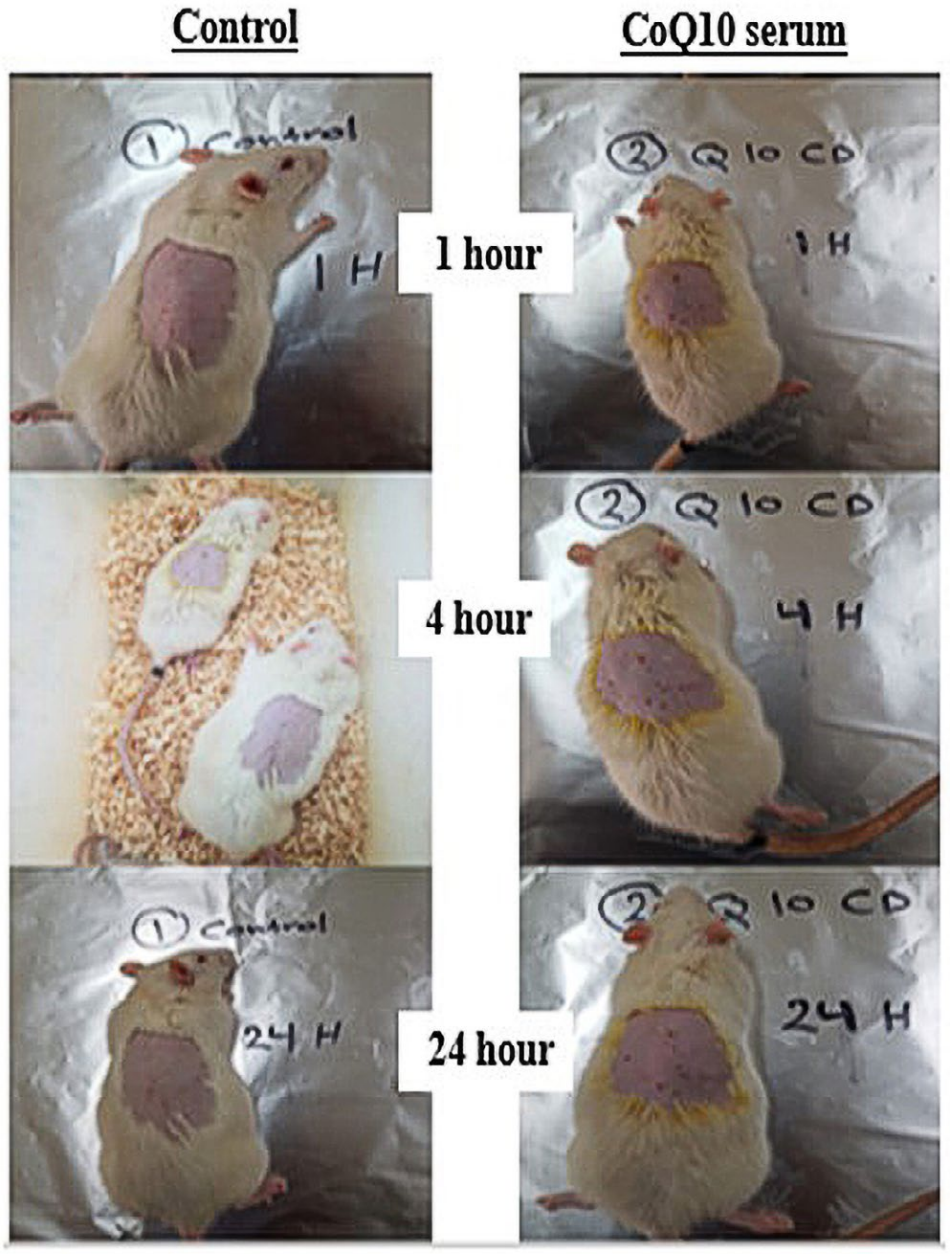
Visual assessment of dermal responses following topical application of CoQ10 serum over a 24‐h period.

An irritation test was undertaken to evaluate the potential reactivity of the CoQ10 serum formula when applied topically. Observations for potential irritation signs were undertaken at multiple post‐application time‐points: 4 min, 1 h, 4 h, and 24 h. The evaluation criteria were based on the dermal Draize irritation scoring system [28] as described in Tables 3 and 4, a reputable system employed to assess the irritative potential of topical agents on animal models. At all the time intervals inspected, the rats exhibited no discernible signs of irritation, erythema, or redness for upon treatment with the CoQ10 serum formula for 24 h (Figure 9). This observation remained consistent upon multiple applications, indicating the serum's potential compatibility and gentleness when applied on the skin. The absence of irritation by the CoQ10 serum formula, even after extended exposure, suggested its potential safety for human application. This is particularly encouraging since many topical products can cause transient irritation, especially in a sensitive location post fur removal.

**TABLE 4:** Dermal responses observed in individual rats showing no signs of any level of erythema and edema by the tested animals.

Wistar Rat (1) control, Rat (2) test	Evaluation after application of test substance			
	0 min	4 min	1 h	24 h
**Erythema**				
(1) Control	0	0	0	0
(2) Test	0	0	0	0
**Edema**				
(1) Control	0	0	0	0
(2) Test	0	0	0	0

Furthermore, CoQ10, also known as ubiquinone, is a naturally occurring antioxidant in the human body. Its well‐documented dermatological benefits range from anti‐aging effects to its potential in increasing skin's resilience. However, it is essential to recognize that the overall irritation potential of a formula is determined not just by its active ingredient but also by its entire composition. The results from the irritation test can be crucial for dermatologists and skincare experts in advocating its safe application, especially for those with sensitive skin. Such findings also pave the way for human trials, which will further validate the serum's compatibility with human skin and showing the safety of CoQ10‐based formulations.

### 3.5 The Stability Studies

Stability testing is paramount in skincare and pharmaceutical industry and when introducing a new formulation. It ensures that the product remains both safe and effective during its shelf life. According to Farough et al. [36], the stability of active ingredients like CoQ10 in this case is indicative of the formula's robustness and viability for long‐term storage. The stability of the CoQ10 serum was thoroughly assessed over a period of 3 months including both the concentration of CoQ10 and several physical parameters.

At the beginning, following the serum's preparation, the CoQ10 recovery stood at 99.5% ± 0.9%. Over time after 1 month, there was a modest decrease in recovery to 97.5% ± 1.1%. By the end of the 3‐month period, the recovery rate showed a slight increase at 98.6% ± 1.5%. The physical attributes of the serum were also closely monitored as they are equally critical indicators of the formula's stability. For instance, changes in viscosity can affect the application, while pH shifts can influence skin compatibility. Throughout the entire 3‐month evaluation, there were no detectable changes in the serum's color, consistency, viscosity, spreadability, and pH. This consistency in physical parameters provided evidence of the serum's stability.

## 4 Conclusion

The complexation of Ubiquinol (CoQ10) with HPβCD was successful in increasing the water solubility remarkably making it easy to be incorporate in hydrophilic formula. The new CoQ10 serum formula has provided valuable insights into its properties and potential in skincare. Key findings highlight the serum's shear‐thinning behavior, which ensures ease of application and high‐quality performance. Its slightly acidic pH, closely aligned with the skin's natural pH, may help enhance the skin's barrier function. The formula demonstrated remarkable stability over 3 months, maintaining consistency in color, viscosity, spreadability, and pH, along with a stable CoQ10 recovery rate. Nonirritation results from animal testing confirmed its safety for skin use. Additionally, the serum showed excellent diffusion properties, suggesting improved skin penetration and effective delivery of active ingredients.

## Author Contributions

All authors have contributed significantly to writing, review, and editing the manuscript and in agreement with the contents. Also, the contents in the manuscript have not been published or submitted for publication elsewhere. Conceptualization and supervision: Maha N. Abu Hajleh and Emad A. Al‐Dujaili; methodology: Hawazin Arkan Yousif, Israa Al‐Ani; and writing and revision: Maha N. Abu Hajleh, Sina Matalqah, and Wael Abu Dayyih. All authors have read and agreed to the published version of the manuscript.

## Conflicts of Interest

The authors declare no conflicts of interest.

## Data Availability

The data that support the findings of this study are available from the corresponding author upon reasonable request.
